# Advancing CAR-T therapy in prostate cancer: overcoming the tumor microenvironment and enhancing efficacy

**DOI:** 10.3389/fonc.2026.1659869

**Published:** 2026-02-04

**Authors:** Zhongze Zhou, Yongfeng Lao, Kun Zhao, Long Cheng, Xi Xiao, Wenxuan Li, Shuai Liu, Xiangbin Kong, Zhilong Dong

**Affiliations:** 1 Department of Urology, Second Hospital of Lanzhou University, Lanzhou, Gansu, China; 2 The Second Hospital & Clinical Medical School, Lanzhou University, Lanzhou, Gansu, China

**Keywords:** CAR design, chimeric antigen receptor T-cell, immunotherapy, prostate cancer, tumor microenvironment

## Abstract

**Background:**

Prostate cancer (PCa) is one of the most common malignancies in men, and metastatic castration-resistant PCa (mCRPC) has limited treatment options. While chimeric antigen receptor T (CAR-T) therapy has revolutionized treatment of hematologic cancers, its efficacy in PCa is constrained by factors such as scarce tumor-specific antigens, an immunosuppressive tumor microenvironment (TME), antigen heterogeneity, and safety issues (e.g., cytokine release syndrome).

**Methods:**

We performed a comprehensive literature review of CAR-T therapy in PCa. We summarized known PCa-specific CAR targets, identified major TME-related and technical barriers, and highlighted recent advances in CAR engineering (including armored CAR-T cells, gene editing, and metabolic reprogramming) as well as combination approaches with other therapies.

**Results:**

Emerging strategies show promise for overcoming these obstacles. Next-generation CAR designs, such as cytokine-armed CAR-T cells, may enhance T cell infiltration and persistence despite the suppressive TME. Modulating tumor metabolism and immune checkpoints can reverse T cell exhaustion. Multi-antigen CARs and targeted gene edits (for example, PD-1 disruption) may limit antigen escape. Early clinical trials in PCa have demonstrated CAR-T cells specifically recognizing prostate-associated antigens and eliciting antitumor immune responses, although durable remissions remain rare.

**Conclusion:**

CAR-T therapy for prostate cancer is a rapidly advancing field. This review provides an updated perspective on CAR-T targets, engineering strategies, and combination approaches in PCa. Ongoing innovations in CAR design and therapeutic combinations offer the potential to develop more effective and durable CAR-T treatments for advanced prostate cancer.

## 1 Introduction

Prostate cancer (PCa) is one of the most commonly diagnosed malignancies and ranks as the second leading cause of cancer-related death among men worldwide (1). According to the 2024 Cancer Statistics report published in *CA: A Cancer Journal for Clinicians*, PCa accounted for an estimated 299,010 new cases in the United States, representing 30% of all male cancers, with approximately 35,250 related deaths projected (1). Its global incidence has steadily increased, posing a significant public health concern. Clinically, PCa progresses from localized tumors to metastatic castration-sensitive disease and ultimately evolves into metastatic castration-resistant prostate cancer (mCRPC), a stage associated with poor prognosis and limited treatment options. Current first-line strategies for advanced PCa include taxane-based chemotherapy, androgen receptor (AR) pathway inhibitors, immune checkpoint inhibitors (ICIs), and the therapeutic cancer vaccine Sipuleucel-T (2). However, these approaches often fail to achieve durable remission in mCRPC, highlighting the urgent need for novel therapeutic strategies.

Adoptive cell therapy (ACT), involving the *ex vivo* expansion and reinfusion of autologous immune cells, has emerged as a promising approach in cancer immunotherapy (3). Among ACT modalities—such as tumor-infiltrating lymphocytes (TILs), T cell receptor-engineered T cells (TCR-T), and chimeric antigen receptor T (CAR-T) cells (4, 5)—CAR-T therapy has demonstrated notable clinical success, particularly in hematologic malignancies including acute lymphoblastic leukemia (ALL) and B cell lymphomas (6). Structurally, CARs consist of an extracellular antigen-binding domain, a hinge/spacer region, a transmembrane domain, and one or more intracellular signaling motifs (7). A key advantage of CAR-T cells is their ability to recognize tumor antigens independently of major histocompatibility complex (MHC), enabling direct T cell activation and cytotoxic responses (7).

Despite these advantages, CAR-T therapy remains clinically challenging in solid tumors like PCa (6, 8). Key barriers include the immunosuppressive tumor microenvironment (TME), tumor antigen heterogeneity, limited CAR-T cell infiltration, and insufficient *in vivo* persistence. Abnormal vasculature and immune exclusion further hinder CAR-T cell trafficking and intratumoral penetration (6, 8–10). To overcome these obstacles, recent studies explore novel tumor-associated antigen (TAA) targets, advanced gene engineering tools, and combinatorial regimens (e.g., CAR-T cells with ICIs, chemotherapeutics, or metabolic modulators).

PCa is considered an immunologically “cold” tumor, partly explaining the limited efficacy of ICIs (11). Nevertheless, CAR-T therapy has garnered increasing interest for PCa treatment, particularly following early-phase clinical trial results (12). These trials confirmed CAR-T cells can recognize prostate-associated antigens and elicit antitumor immunity. However, immunosuppressive TME components, T cell exhaustion, and antigen escape continue to hinder long-term success (13).

In this review, we comprehensively overview the CAR-T therapy landscape in prostate cancer, highlighting emerging targets, structural/functional CAR modifications, TME challenges, and advances in armored CARs, metabolic reprogramming, and combination strategies. Our goal is to clarify CAR-T therapy’s translational potential in PCa and guide innovations toward effective, durable clinical applications.

## 2 CAR-T cell design

### 2.1 CAR-T cell structure and classification

CAR-T cells are genetically engineered T lymphocytes that express a synthetic chimeric antigen receptor (CAR) (14, 15). The CAR structure typically consists of three key components: (i) an extracellular domain, (ii) a transmembrane domain, and (iii) an intracellular signaling domain (14, 15). The extracellular domain comprises a single-chain variable fragment (scFv) that specifically recognizes tumor-associated antigens (TAAs). The transmembrane domain anchors the receptor in the T cell membrane and is commonly derived from proteins such as FcϵRIγ, CD3ζ, CD8α, or CD28 (15). The intracellular domain contains immunoreceptor tyrosine-based activation motifs (ITAMs), responsible for signal transduction and T cell activation. These motifs are commonly derived from the CD3ζ chain of the T cell receptor complex or the FcϵRI γ chain (16).

**Figure 1:**
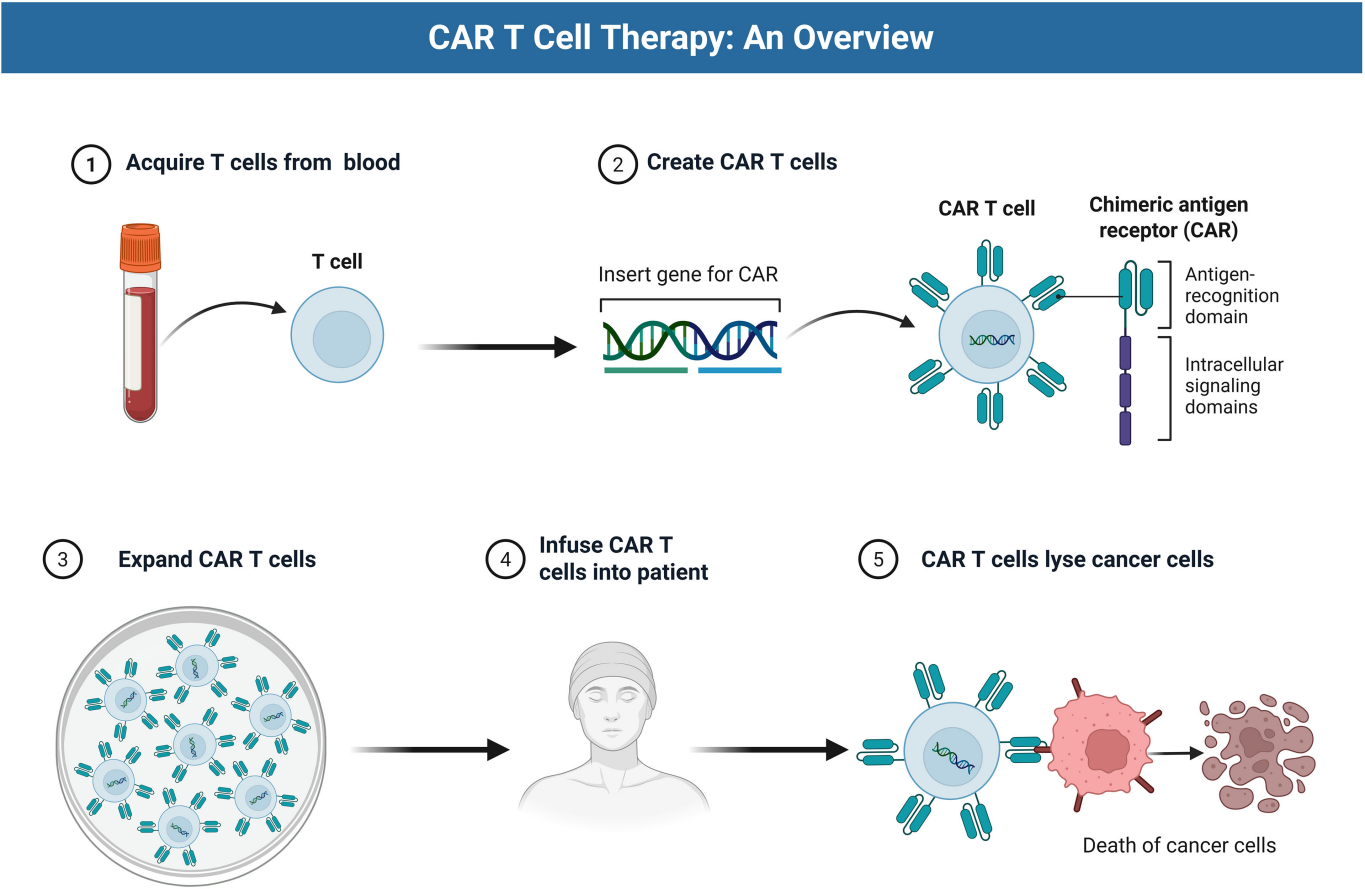
Workflow of CAR‐T cell therapy. This schematic illustrates the clinical process of CAR‐T therapy. First, patient T lymphocytes are harvested and genetically engineered ex vivo to express a chimeric antigen receptor (CAR) – a synthetic receptor composed of an extracellular single‐chain variable fragment (scFv) for tumor‐antigen binding, a hinge/transmembrane region, and intracellular signaling domains (e.g. CD3ζ ITAM). The modified T cells are expanded in culture and then re-infused into the patient. Once *in vivo*, CAR‐T cells home to the tumor, recognize tumor‐associated antigens via the CAR scFv domain, and become activated through the receptor’s intracellular motifs. Activated CAR‐T cells proliferate and kill cancer cells (e.g. by inducing tumor‐cell apoptosis), leading to tumor regression.

As shown in **Figure 1**, CAR-T manufacturing involves leukapheresis, engineering, expansion, and reinfusion.

### 2.2 Evolution of CAR-T cell generations

First-generation CAR-T cells incorporate only the CD3ζ chain as their intracellular signaling domain, lacking costimulatory motifs (17). Consequently, they undergo rapid post-infusion apoptosis, display limited antitumor activity, poor persistence, and reduced expansion *in vivo*. Second-generation CAR-T cells introduce an additional costimulatory domain (e.g., CD28 or 4-1BB) alongside CD3ζ (18, 19). This modification enhances proliferation, cytokine secretion, survival upon antigen engagement, and overall antitumor efficacy (18, 19). These constructs are widely used clinically, particularly for hematologic malignancies.

Third-generation CAR-T cells integrate two costimulatory domains, such as OX40 (CD134) or 4-1BB (CD137), with CD3ζ. Although they exhibit enhanced cytotoxicity and antitumor effects in preclinical studies, third-generation CAR-T cells have not yet demonstrated significant clinical benefits. Fourth-generation CAR-T cells, termed T cells redirected for universal cytokine-mediated killing (TRUCKs), incorporate transgenes encoding proinflammatory cytokines (e.g., IL-12, IL-15) to enhance immune responses (20). These cytokines recruit and activate immune cells, overcoming the immunosuppressive tumor microenvironment (21).

**Figure 2:**
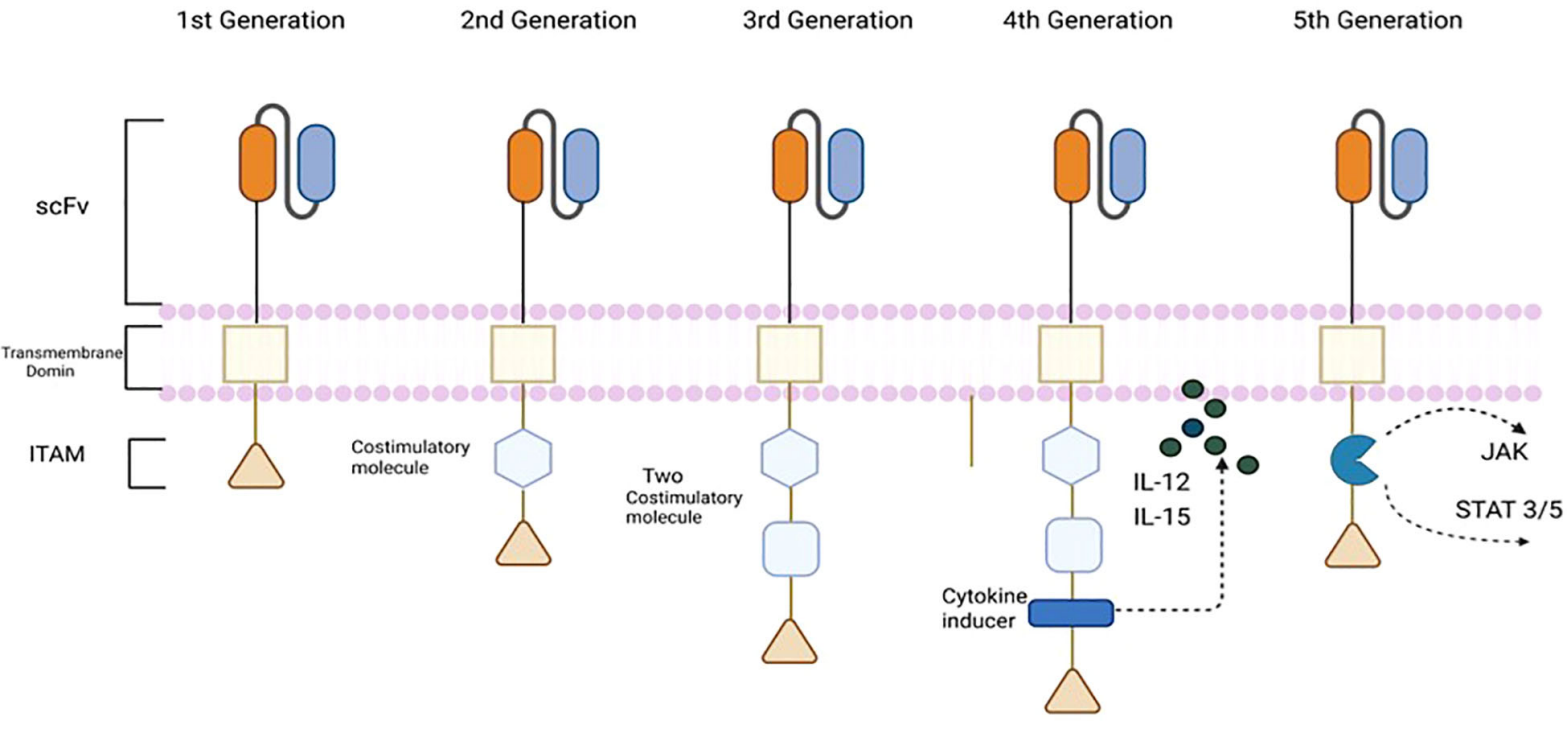
Structural evolution of CAR constructs by generation. This diagram compares first through fifth generation CAR designs. First‐generation CARs contain only the CD3ζ signaling chain (with its immunoreceptor tyrosine‐based activation motifs, ITAMs) and no costimulatory domain. Second‐generation CARs add a single costimulatory domain (such as CD28 or 4-1BB) alongside CD3ζ, markedly enhancing T‐cell proliferation and persistence. Third‐generation CARs incorporate two costimulatory motifs (for example, CD28 plus OX40 or 4-1BB) to further boost cytotoxicity. Fourth‐generation CARs (so‐called “TRUCKs”) include an additional transgene encoding a pro‐inflammatory cytokine (e.g. interleukin-12 or interleukin-15) to recruit and activate other immune cells. Fifth‐generation CARs fuse a truncated IL-2 receptor β-chain domain that activates the JAK–STAT3/5 pathway upon antigen engagement, enabling simultaneous signaling through CD3ζ, costimulatory domains, and cytokine pathways.

Fifth-generation CAR-T cells incorporate a truncated IL-2 receptor β-chain domain that engages the JAK-STAT3/5 pathway (22). This design enables simultaneous activation of CD3ζ, a costimulatory signal (e.g., CD28), and the JAK-STAT axis upon antigen engagement, enhancing T cell activation, proliferation, and function (22) (**Figure 2**).

### 2.2 Gene transfer methods

CAR transgenes can be introduced into T cells through viral or non-viral systems. In clinical practice, gamma retroviruses and lentiviral vectors are most widely used due to their stable genome integration and high transduction efficiency; However, they require GMP vector production, copy number control, and replication competent virus (RCL) testing, and they have a lower risk of insertion mutagenesis (vector design can mitigate this) (23). non-viral methods include transposons (Sleeping Beauty or PiggyBac), which can be integrated without viral production but may produce more variable copy numbers and typically require electroporation/longer culture times (24, 25), as well as CRISPR/Cas mediated site-specific knockouts (such as TRAC) (26), which provide predictable insertion sites and reduced tonic signaling, but at the cost of reduced efficiency, double-strand breaks, and increased QC. mRNA electroporation produces transient CAR expression, which can be used for early safety exploration, but typically requires repeated administration to maintain activity.

### 2.3 Manufacturing considerations

Building on gene transfer methods, feasibility is ultimately shaped by vein-to-vein logistics, platform-specific QC/release testing (e.g., viability/phenotype; sterility/mycoplasma/endotoxin; VCN/RCL for viral products; editing-specific off-target/translocation QC), and turnaround time (see **Figure 1** for the workflow) (27). In mCRPC, real-world cohorts consistently show a median overall survival of only around two to three years from diagnosis, and a substantial proportion of patients never receive second-line life-prolonging therapy because of age, comorbidities and functional decline (28, 29). In this setting, an autologous CAR-T manufacturing process with a vein-to-vein time of 3–5 weeks means that some patients will deteriorate or become ineligible between leukapheresis and infusion. Manufacturing strategies that reduce turnaround time or enable off-the-shelf products are therefore a key component of the practical feasibility of gene-engineered cell therapies in mCRPC.

## 3 Prostate cancer tumor microenvironment

### 3.1 Composition of the TME in prostate cancer

The tumor microenvironment (TME) of prostate cancer forms a critical ecological niche that supports tumor survival, progression, and adaptation (30) and comprises immune cells, stromal cells, vascular endothelial cells, and a complex network of cytokines and signaling molecules. The TME drives tumorigenesis, progression, metastasis, and treatment resistance in prostate cancer. Its profoundly immunosuppressive nature poses a major barrier to effective immunotherapy (31).

### 3.2 Function of the TME in prostate cancer

#### 3.2.1 Immunosuppressive cell populations

The tumor microenvironment (TME) of prostate cancer contains diverse immunosuppressive cell populations, including tumor-associated macrophages (TAMs), myeloid-derived suppressor cells (MDSCs), and regulatory T cells (Tregs). Among them, Tregs suppress immune responses by inhibiting effector T cells and natural killer (NK) cells via cell–cell contact and secretion of inhibitory cytokines (e.g., IL-10, TGF-β). Tregs are elevated in prostate tumors and correlate with poor prognosis (32).

Myeloid suppressor cells (MDSCs) are abundant in prostate TME and exhibit heterogeneity in phenotype and function. Traditionally, they suppress anti-tumor immunity by releasing cytokines such as ARG1, reactive oxygen species/nitrogen, and IL-23, thereby inhibiting T cell proliferation, promoting Treg expansion, and contributing to resistance to androgen deprivation therapy. However, depending on the organizational environment, activation status, and cytokine environment, MDSCs can also suppress immunity through pro-inflammatory means, such as producing IL-1β/IL-6/IL-23, thereby inhibiting Th17 response and reshaping the TME. This duality makes MDSC targeted interventions complex and should be considered when integrating T cell targeted therapy. Meanwhile, M2 polarized tumor associated macrophages (TAMs) secrete tumor promoting factors (such as VEGF, IL-6) (33), activate STAT3 signaling, promote angiogenesis, inhibit effector T cells, and aid in tumor progression and metastasis (34).

#### 3.2.2 Cytokines and checkpoint molecules

T-cell exhaustion is another hallmark of the prostate cancer TME. CD8^+^ T cells frequently upregulate PD-1, TIM**-**3, and LAG**-**3, which is associated with functional attrition (35). In parallel, Th1 responses are attenuated, whereas Th2/Th17 polarization predominates (36). Th2 cytokines impair cellular and humoral immunity (37), and Th17/IL-17–driven inflammation can support tumor progression (35). B cells play dual roles: although they can present antigen, Bregs (e.g., IL-10^+^ subsets) promote Treg activity and immunosuppression, sometimes correlating with worse outcomes (35, 38). NK-cell cytotoxicity is blunted—partly due to reduced IL-12 from TAMs and TGF-β–mediated down-modulation of NKG2D (39). DCs are fewer and less mature, compromising priming and, by extension, CAR-T persistence (40). Finally, immunosuppressive cytokines shape the milieu: IL-6/STAT3 dampens antitumor immunity, IL-8/STAT3–MALAT1 supports tumor growth, and TGF-β drives Treg differentiation while restraining effector T/NK function—directly limiting CAR-T activity; IL-23 and IL-17 further perpetuate chronic inflammation and immune escape (13, 35, 37, 41).

#### 3.2.3 Metabolic modulation and immune evasion

##### 3.2.3.1 Chemokine-mediated immunosuppression

Cancer-associated fibroblasts (CAFs) secrete CXCL12, recruiting immunosuppressive cells (42). Tumor-associated macrophages (TAMs) release CCL2 and CXCL8, promoting their own recruitment and M2 polarization (43), and activate signal transducer and activator of transcription 3 (STAT3)/metastasis-associated lung adenocarcinoma transcript 1 (MALAT1) and mitogen-activated protein kinase/extracellular signal-regulated kinase (MAPK/ERK) pathways, driving tumor growth and therapy resistance (43). M2-polarized TAMs release CXCL5 and CCL6, facilitating immunosuppression and bone metastasis (44). CXCR2-recruited myeloid-derived suppressor cells (MDSCs) secrete interleukin-10 (IL-10) and transforming growth factor-beta (TGF-β), expanding regulatory T cells (Tregs) and suppressing T cells (45). Tregs secrete inhibitory cytokines and express checkpoint ligands, suppressing CD8^+^ T cell responses (46).

##### 3.2.3.2 Checkpoint molecule dysregulation

Multiple inhibitory receptors—including programmed cell death protein 1 (PD-1), cytotoxic T-lymphocyte-associated protein 4 (CTLA-4), lymphocyte-activation gene 3 (LAG-3), and T cell immunoglobulin and mucin-domain containing-3 (TIM-3)—are expressed, contributing to poor T cell infiltration and exhaustion (47). These molecules induce T cell anergy, impairing antigen recognition. TAMs and Tregs express programmed death-ligand 1 (PD-L1) and other checkpoint ligands, secreting IL-10 and TGF-β (39)to further impair effector T cells (48). TAM-expressed PD-1 may attenuate antitumor activity and neutralize anti-PD-1 therapy via antibody scavenging (49).

The TME is metabolically rewired. Lactate from aerobic glycolysis sustains Tregs under acidosis and skews macrophages toward an M2 program, suppressing CD8^+^ T cells (39). Arginase-1 expressed by TAMs depletes arginine, destabilizing CD3ζ and blunting cytotoxicity (50), while IDO-driven tryptophan catabolism generates immunosuppressive metabolites that inhibit T cells and expand Tregs (42). In this context, fatty acids fuel FAO/OXPHOS in Tregs and M2-TAMs (51), cholesterol perturbs membrane signaling and activation (52), and MDSC-derived PGE_2_ impairs DC maturation and T-cell function (53). Together, these shifts promote T-cell exhaustion/dysfunction and resistance to checkpoint blockade (54–56). Targeting such pathways—for example, with an EP4 antagonist (YY001)—shows promise for restoring T-cell infiltration and enhancing immunotherapy (57).

#### 3.2.4 Strategies to remodel the TME

In summary, the profoundly immunosuppressive tumor microenvironment (TME) in prostate cancer results from synergistic interactions among suppressive cells, effector cell exhaustion, and dysregulated immunoregulatory mediators. This immunologically “cold” state compromises natural antitumor immunity and impairs chimeric antigen receptor T (CAR-T) cell infiltration and cytotoxicity (58). TME remodeling strategies (e.g., TGF-β blockade, Treg/MDSC depletion, DC activation) may enhance CAR-T efficacy against prostate cancer (59).

## 4 Application of CAR-T cell therapy in prostate cancer

### 4.1 Prostate tumor-associated antigens in CAR-T therapy

Identifying tumor-associated antigens (TAAs) specific to prostate cancer (PCa) is critical for chimeric antigen receptor T (CAR-T) cell therapy development. Ideal targets exhibit high tumor expression with minimal normal tissue distribution to enable robust, specific immune responses. Key PCa TAAs include prostate-specific antigen (PSA), prostate-specific membrane antigen (PSMA), prostatic acid phosphatase (PAP), and prostate stem cell antigen (PSCA).

#### 4.1.1 Prostate-specific antigen

In transgenic mice expressing human PSA and HLA-A2.1, androgen deprivation enhanced PSA-specific cytotoxic T lymphocyte (CTL) responses, suggesting castration-induced immunogenicity (60).

#### 4.1.2 Prostate-specific membrane antigen

PSMA is a type II transmembrane glycoprotein (glutamate carboxypeptidase II) that is highly expressed on prostate epithelium and upregulated in prostate cancer, where higher levels correlate with Gleason grade, castration resistance, and tumor-associated neovascularization (60, 61). PSMA-targeted PET imaging (e.g., ^68Ga-PSMA-11) is now routine for detection and recurrence work-up, underscoring the antigen’s consistent tumor expression; nevertheless, low-level expression in salivary glands and renal tubules raises on-target/off-tumor considerations (60, 61).Foundational work by Maher et al., 2002 showed that PSMA-redirected human T cells (TCRζ/CD28) selectively lysed PSMA^+ prostate lines and proliferated upon antigen engagement (62). Building on this, Gade et al., 2005 demonstrated eradication of prostate tumors across subcutaneous, orthotopic, and pulmonary metastasis models using PSMA-targeted T cells (63). Zuccolotto et al., 2014 reported that a second-generation PSMA CAR (CD28 costimulation) produced near-complete regression and survival benefit in disseminated xenografts (64); armoring to resist the prostate TME—for example a dominant-negative TGF-β receptor—further enhanced antitumor activity (65). In addition, Alzubi et al., 2020 showed that non-ablative low-dose docetaxel synergized with PSMA CAR-T, supporting the rationale for chemotherapy-assisted regimens in preclinical prostate models (66). Collectively, preclinical data support antigen-specific efficacy and provide engineering levers (costimulation, TGF-β resistance, and rational combinations). In an early first-generation study, Junghans et al., 2016 observed PSA declines of ≥50% and ≥70% in 2/5 men with mCRPC, establishing feasibility but limited persistence (67). More recently, Slovin et al., 2022 reported a phase-1 experience with P-PSMA-101 (a piggyBac^®^-engineered autologous PSMA CAR-T), noting manageable safety, PSA declines ≥30% in a subset of patients, and variable expansion/durability (68). Overall, PSMA remains one of the most clinically advanced targets for prostate-directed CAR-T therapy.

Most PSMA CAR-T preclinical studies, including those from Maher, Gade and Zuccolotto, use xenograft models with uniform, high PSMA expression in severely immunodeficient mice, which tend to overestimate antitumor activity and do not capture antigen heterogeneity, bone metastases or intact immune regulation seen in patients. By contrast, early clinical trials of PSMA-directed CAR-T cells in mCRPC, such as the piggyBac-derived product P-PSMA-101 and the TGFβ-insensitive construct CART-PSMA-TGFβRDN, have shown PSA and radiographic responses only in a subset of heavily pretreated patients, with variable durability and cytokine-driven toxicities. The discrepancy between the near-complete tumor control in mouse models and the modest clinical benefit observed so far most likely reflects limited *in vivo* persistence and trafficking of PSMA CAR-T cells within an immunosuppressive, bone-predominant tumor microenvironment, together with late-line patient selection and heterogeneous PSMA expression.

#### 4.1.3 EpCAM (CD326)

EpCAM (CD326) is a transmembrane glycoprotein overexpressed across epithelial malignancies, including prostate cancer. Although constitutively present on normal epithelia, dysregulated expression in cancer stem–like and malignant cells promotes progression and metastasis—partly by weakening E-cadherin–mediated adhesion and enhancing proliferation, migration, and differentiation (69). Its frequent, high-level expression makes EpCAM an attractive yet safety-sensitive immunotherapeutic target because of potential on-target/off-tumor activity in healthy epithelia.Ni et al. showed that shRNA knockdown of EpCAM in PC-3 xenografts increased radio- and chemosensitivity, with immunohistochemistry indicating down-regulation of PI3K/AKT/mTOR signaling—relevant to CRPC biology (70). Deng et al. engineered EpCAM-targeted CAR-T cells that killed PC-3/PC-3M *in vitro* and, in mice, significantly suppressed primary tumors and reduced lung/bone metastases, yielding 100% 80-day survival versus 33% in controls (71).Because EpCAM is broadly expressed on normal epithelia, clinical translation will likely require strategies to mitigate off-tumor toxicity (e.g., affinity tuning, logic-gated CARs, local or regional delivery, and safety switches), and prospective studies are needed to define therapeutic window and durability. Because EpCAM is physiologically expressed on normal epithelia—including airway/alveolar and gastrointestinal epithelium—there is a risk of on-target/off-tumor toxicity; in mice, high-dose EpCAM-CAR-T provoked dose-dependent lung injury (72). Prognostically, EpCAM shows context-dependent value in prostate cancer: large tissue-microarray work found no independent correlation with Gleason grade or survival (73), underscoring inter-tumoral heterogeneity and the need for biomarker-driven selection. Early-phase, open-label studies of EpCAM-directed approaches in EpCAM-positive solid tumors are ongoing—e.g., NCT03013712 and NCT02915445—with correlative biomarker analyses that may include prostate cohorts; however, prostate-specific efficacy data remain limited (74).

Clinically, EpCAM expression on normal epithelial and gastrointestinal tissues has translated into dose-limiting toxicity for several systemic EpCAM-directed agents, including immunotoxins and bispecific antibodies, which has constrained both the achievable dose and the feasibility of repeated systemic schedules. In contrast, some of the more successful programs, such as intraperitoneal catumaxomab for malignant ascites, have deliberately used regional administration to confine drug exposure to the peritoneal cavity and keep systemic levels low. In light of these safety constraints, together with the imperfect and sometimes unstable prognostic information derived from EpCAM-based CTC assays, EpCAM appears more suitable as a target for regionally delivered or affinity-tuned approaches than for high-dose systemic CAR-T or T-cell–engaging therapies.

#### 4.1.4 Prostatic acid phosphatase

PAP is a glycoprotein with tyrosine-phosphatase activity secreted by benign and malignant prostate epithelium. Its expression tends to decrease with increasing tumor grade, being highest in Gleason 6–7 tumors. PAP gained clinical prominence through sipuleucel-T (Provenge^®^), which primes autologous antigen-presenting cells ex vivo with PA2024 (a recombinant PAP/GM-CSF fusion) to elicit PAP-specific T-cell responses. In the phase III IMPACT trial, sipuleucel-T reduced the risk of death by 22.5% and prolonged median overall survival by 4.1 months (25.8 vs. 21.7 months) (75). Immune-related adverse events were generally low-grade and included chills, fever, and headache in >20% of patients (76). While IMPACT validates PAP as an immunogenic antigen, it does not establish PAP as an optimal CAR-T target. CAR-T cells require abundant, accessible cell-surface antigen, whereas PAP is primarily secreted/intracellular, and its tumor-cell surface density appears limited—features that may blunt CAR engagement despite vaccine responsiveness. Moreover, PAP expression declines in high-grade and castration-resistant disease (77), and extra-prostatic expression (kidney, testis, placenta and several non-prostate malignancies) raises on-target/off-tumor concerns (77, 78). Pathologically elevated serum PAP—for example during tissue injury—could act as an antigen sink and potentially increase systemic exposure/toxicity risk (79). Taken together, PAP is a clinically validated vaccine antigen with proven survival benefit, but as a CAR-T target it faces constraints in antigen accessibility, grade-dependent down-regulation, and normal-tissue expression. A more rational direction for future work may be to further develop PAP-based vaccination strategies in combination with other immunomodulatory approaches, rather than to continue investing heavily in PAP-CAR-T constructs.

#### 4.1.5 Prostate Stem Cell Antigen (PSCA)

PSCA is a GPI-anchored glycoprotein expressed in normal prostate epithelium and overexpressed in >80% of prostate cancers—most prominently in metastatic disease and higher Gleason grades—where expression tracks with progression and bone involvement (79). Multiple groups have shown that PSCA-directed CAR-T cells elicit antigen-specific cytokine release and cytolysis against PC-3, LAPC-9, and DU145 *in vitro*, suppress tumor growth, prolong survival, and in some immunodeficient mouse models produce complete regressions (80). Hillerdal et al., 2014 reported that third-generation PSCA CAR-T cells increased IL-2/IFN-γ secretion, expanded CTLs, and improved tumor control; inclusion of costimulatory domains such as 4-1BB further enhanced cytotoxicity and persistence (80). Bispecific constructs co-targeting PSCA/PSMA reduced antigen-escape in preclinical systems (80).

In skeletal models, systemically administered PSCA CAR-T cells trafficked to intratibial tumors and limited disease (Priceman et al., 2017) (81). To counter PD-1/PD-L1–mediated inhibition, PD-1–silenced PSCA CAR-T cells showed superior *in-vivo* activity (Zhou et al., 2021) (82). Clinical experience (NCT03873805). In a phase I study in mCRPC, Dorff et al., 2024 reported ≥30% PSA declines in 4/14 patients (including one >90%), radiographic reductions in tumor burden, and a correlation between effector-memory T-cell expansion and response (12).

Overall, PSCA is prevalent in prostate cancer and biologically plausible for CAR-T. However, early clinical signals indicate limited *in-vivo* persistence and on-target/off-tumor cystitis as a potential dose-limiting toxicity, suggesting a narrow safety window. Next steps include fractionated dosing and persistence-oriented engineering (e.g., 4-1BB costimulation, checkpoint modulation), rational combinations (e.g., RT priming), and biomarker-enabled phase I/II trials to quantify durable benefit relative to PSMA-directed approaches.

#### 4.1.6 Emerging target: STEAP1

Six-transmembrane epithelial antigen of the prostate-1 (STEAP1) shows increased expression in prostate cancer and correlates with adverse features, including higher Gleason score and serum PSA levels (83, 84). Jin et al. generated a STEAP1-specific monoclonal antibody and engineered second-generation STEAP1 CAR-T cells that lysed 22Rv1, LNCaP, and C4-2B prostate cancer cells and secreted high levels of IFN-γ and TNF-α upon antigen engagement (85). Target dependence was confirmed by STEAP1 knockdown, which diminished CAR-T responsiveness and, independently, reduced dihydrotestosterone (DHT)–stimulated proliferation, reinforcing the biological relevance of STEAP1 in androgen-driven disease (86). Sasaki et al. developed a less toxic and more effective STEAP1 CAR-T cell therapy by fusing IL-12 with collagen binding domains, activating the host immune system and combating antigen heterogeneity. It has shown good therapeutic effects in both mouse and human prostate cancer models (87). These data support STEAP1 as an emerging prostate cancer antigen for cellular therapies; however, clinical activity has yet to be defined. Early-phase studies should incorporate biomarker-based selection (tumor STEAP1 density/heterogeneity), persistence-oriented engineering, and careful safety monitoring to delineate the therapeutic window, potentially alongside androgen-axis modulation given the DHT link (83, 85, 86).

#### 4.1.7 B7-H3 (CD276)

B7 homolog 3 protein (B7-H3/CD276) is overexpressed in prostate cancer tissues and cell lines (e.g., PC-3, DU145, LNCaP) compared to benign prostate epithelium (88, 89). Building on this biology, Li et al. engineered second-generation B7-H3 CAR-T cells that mediated antigen-specific cytotoxicity against B7-H3^+^ prostate cancer cells without lysis of B7-H3^-^ controls, accompanied by robust IFN-γ/TNF-α secretion on engagement. In DU145 xenografts these cells produced >70% tumor-growth inhibition (P<0.01 vs controls) with no apparent toxicity on body weight or organ histology (90). These data indicate target dependence and an initial preclinical safety window. Although B7-H3 is frequently tumor-associated, low-level expression on some normal epithelia/vasculature raises potential on-target/off-tumor concerns; affinity tuning, logic-gated designs, or safety switches may therefore be advisable in early human testing. Phase I studies of B7-H3 CAR-T cells in solid tumors are ongoing—e.g., NCT04864821—with prostate-specific cohort readouts still pending. Defining epitope density/heterogeneity in metastatic sites and establishing persistence will be key to determine clinical benefit in prostate cancer.

B7-H3 has a comparatively mature clinical portfolio in solid tumors, with multiple phase I/II studies of monoclonal antibodies, antibody–drug conjugates and CAR-T cells, while prostate cancer-specific CAR-T experience is limited to small early-phase cohorts or ongoing trials. At this stage, B7-H3 probably merits inclusion of prostate cancer within broader pan–solid-tumor B7-H3 CAR-T platforms, but the data remain too preliminary to justify launching a dedicated, large-scale B7-H3 CAR-T program focused solely on mCRPC.

#### 4.1.8 Ephrin Type-A receptor 2 (EphA2)

Ephrin type-A receptor 2 (EphA2) is overexpressed in metastatic prostate cancer and correlates with poor prognosis (91, 92). Its expression extends to breast and cervical malignancies, suggesting pan-cancer therapeutic potential (93). Building on this rationale, Zhang et al. engineered a second-generation EphA2 CAR incorporating an EphA2-specific scFv (clone not specified), a CD8α hinge/transmembrane segment, CD28 costimulation, and a CD3ζ signaling module (92). *In vitro*, these CAR-T cells achieved >95% lysis of PC-3, DU145, and LNCaP at an E:T of 5:1 within 120 h, underwent antigen-dependent proliferation (~3.2-fold vs controls), and secreted high IFN-γ (1,842 ± 206 pg/mL) upon engagement (92). In DU145 xenografts, treatment produced ~78% tumor-growth inhibition (P < 0.001) relative to untransduced T cells; after a second infusion, 5/8 mice achieved complete regression. Throughout dosing, body weight remained stable (± 5%), serum ALT/AST were within normal range, and no CRS-like signs were observed (92). EphA2 has shown good preclinical efficacy in prostate models. Early design should consider affinity tuning or logic gated CAR, cautious dose segmentation and lung monitoring (symptoms, blood oxygen saturation, liver enzymes), and biomarker based selection (EphA2 density/heterogeneity at lesion level) to position EphA2 as a supplementary option, especially in PSMA low disease.

#### 4.1.9 Human kallikrein-related peptidase 2 (hK2/KLK2)

Human kallikrein-related peptidase 2 (hK2/KLK2) is a prostate-restricted serine protease with high expression in malignant prostate epithelium and limited distribution in normal tissues. The first bispecific T-cell junction antibody Pasritamig (KLK2 × CD3) targeting KLK2 was included in a phase I study of 174 previously treated mCRPC patients; The determined RP2D is 3.5 mg (D1) → 18 mg (D8) → 300 mg (once every 6 weeks from D15, intravenous), with good overall tolerability (94). The main treatment-related adverse events in the RP2D safe population are infusion related reactions, fatigue, and CRS 8.9%/grade 1. In the RP2D efficacy set (n=33), the median rPFS was 7.85 months, and the PSA50 reached 42.4%, demonstrating safe outpatient administration and validating the feasibility and conceptual efficacy of KLK2 as an immunotherapy target for prostate cancer. Follow-on studies are ongoing, including combination trials (NCT06095089; NCT07082920) and a randomized phase 3 study (NCT07164443), supporting the feasibility of targeting KLK2 in prostate cancer.

KLK2 is currently being targeted in prostate cancer by non–cell-based platforms such as the KLK2×CD3 bispecific pasritamig and actinium-labeled KLK2 antibodies, which are in early-phase clinical testing, whereas no KLK2-directed CAR-T trials in humans have been reported so far. As systemic safety and therapeutic index are only now being defined through these bispecific and radioligand programs, KLK2 is not yet a realistic candidate for large, resource-intensive CAR-T projects, but could become attractive once more robust clinical targeting and safety data are available.

### 4.2 Alternative effector platforms (universal T, NK, macrophage)

Beyond autologousαβT cells, several platforms are being explored to address solid-tumor barriers. Allogeneic (“universal”) T cells edited at loci such as TRAC—with or without additional B2M/HLA engineering—can be delivered off-the-shelf and have demonstrated clinical feasibility in early studies (e.g., TALEN-edited UCART19); however, host rejection and the added quality-control (QC) requirements associated with genome editing remain practical constraints (26). CAR-NK cells from cord blood, peripheral blood or iPSCs have produced clinical responses with low rates of CRS/ICANS in B-cell malignancies and are being adapted to prostate antigens; persistence is typically limited and often requires IL-15 support (95, 96). PSMA is currently the only prostate target that has entered a dedicated CAR-NK trial in mCRPC. An anti-PSMA CAR-NK product (TABP EIC) is being evaluated in a first-in-human phase I study in patients with metastatic castration-resistant disease (NCT03692663), with safety and feasibility as the primary endpoints and no peer-reviewed outcome data reported so far. In parallel, several groups have demonstrated that PSMA-CAR-NK-92 or peripheral blood–derived CAR-NK cells can mediate potent (97), antigen-specific cytotoxicity against PSMA-positive CRPC cell lines and control tumor growth in xenograft models, particularly when combined with PD-1/PD-L1 blockade (98). For PSCA, off-the-shelf PSCA-CAR_s15 NK cells and more recent PSCA-CAR-NK constructs have shown strong activity in PSCA-expressing solid-tumor models, and a new study has confirmed cytotoxicity of PSCA-CAR-NK cells against PSCA-positive tumor cell lines and characterized a chemokine receptor profile compatible with solid-tumor trafficking. Taken together, PSMA- and PSCA-directed CAR-NK platforms appear promising enough to justify further, carefully sized early-phase studies in mCRPC, but the evidence is still too limited to support large, registrational CAR-NK programs in this disease.

CAR macrophages (CAR-M) mediate phagocytosis, antigen presentation, and myeloid remodeling in preclinical models (99); first-in-human testing in HER2-positive solid tumors has shown feasibility (100, 101), but manufacturing consistency and *in vivo* durability remain unresolved. PSMA-specific CAR-macrophages have recently been engineered and shown to phagocytose PSMA-positive prostate cancer cells *in vitro* and to inhibit tumor growth in PSMA-expressing xenograft models, while repolarizing the tumor microenvironment toward a more inflammatory phenotype (102). In this context, alternative effector platforms are best viewed as complementary exploratory strategies—particularly for patients who may not be ideal candidates for intensive autologous CAR-T procedures or for rational combinations with other PSMA/PSCA-directed agents—rather than as ready substitutes for T-cell–based CAR therapies in mCRPC at present.

## 5 CAR-T cell therapy-associated adverse effects

### 5.1 Cytokine release syndrome

CRS is the most frequent acute toxicity of CAR-T therapy. In CD19 CAR-T trials for B-cell malignancies, any-grade CRS occurs in ~60–90% of patients, although grade ≥3 events are less common (103, 104). Robust incidence data are not yet available for prostate-directed CAR-T because clinical experience remains limited (105). This systemic inflammatory response results from excessive T cell activation, triggering hyper-elevation of interleukin-6 (IL-6), interferon-gamma (IFN-γ), IL-10, and IL-8 (106).

To manage CRS, clinical practice commonly uses tiered supportive care and pharmacologic interventions. Corticosteroids are a mainstay; when given under protocol-defined indications and dosing, they can rapidly suppress inflammation with generally manageable impact on CAR-T expansion and antitumor activity (103, 107). For steroid-refractory cases, the combination of etanercept (TNF inhibitor) and tocilizumab (anti–IL-6 receptor) has been reported to expedite symptom control and is incorporated into standardized pathways at many centers (107).

In the PSMA-directed CART-PSMA-TGFβRDN phase 1 study, dose-dependent toxicities included high-grade CRS and a fatal case in which grade 4 CRS was followed by severe infection and multiorgan failure despite apparent resolution of the initial cytokine storm. Early experience with P-PSMA-101 and PSMA-targeted bispecifics has likewise shown that substantial CRS can occur in this frail population, leading to protocol amendments and treatment holds. These observations suggest that even “standard” CRS-management algorithms may be more difficult to apply in mCRPC because transfusion support, fluid resuscitation and broad-spectrum antibiotics must be balanced against limited marrow reserve, borderline renal function and a high background risk of skeletal-related events (104). Future PCa-directed CAR-T trials should use more conservative dose escalation and stricter eligibility criteria than those typically applied in younger hematologic cohorts. Practical safeguards include minimum marrow-reserve cut-offs (Hb/platelets adjusted for prior radioligand therapy and marrow involvement), exclusion of uncontrolled renal dysfunction, and structured monitoring of bone disease–related complications during the CRS window; step-up dosing or modified lymphodepletion may be considered for heavily pretreated patients to keep CRS manageable while preserving representativeness. Additional cytokine-directed strategies are under investigation. JAK-1 inhibitors attenuate the JAK–STAT cascade and can reduce the production of multiple inflammatory mediators—including IL-6, IL-12, and IFN-γ—thereby mitigating CRS-related inflammation (108).

CRS manifestations are summarized in **Table 1**.

**Table 1:** Clinical manifestations of cytokine release syndrome (CRS) and corresponding first-line management strategies in CAR-T therapy.

Symptom/sign	Typical onset window	Representative vitals/labs	Grade anchor (ASTCT/CTCAE)	First-line management
Fever, malaise	Day 1–5 post-infusion	IL-6↑, CRP↑	G1–2	Antipyretics; close monitoring
Hypotension	Escalating or persistent	MAP↓, lactate↑	≥G3	Tocilizumab; IV fluids; vasopressors as needed
Hypoxia	With/without infiltrates	SpO_2_↓, ABG abnormal	≥G3	Oxygen support; treat co-infections; consider steroids

### 5.2 On-target, off-tumor toxicity

The expression of several CAR-T cell targets, including PSCA, PSMA, and EpCAM, is not restricted to cancer cells but also occurs in various healthy tissues. This overlap can lead CAR-T cells to inadvertently attack normal tissues, resulting in on-target, off-tumor toxicity. To mitigate this risk, strategies focusing on enhanced specificity are being developed. Yu et al. proposed using bispecific T-cell engagers (BiTEs) that simultaneously target two antigens, reducing off-tumor toxicity while also countering antigen escape (109).

PSMA- or PSCA-directed T cell engagers (including BiTE/TCE formats) are attractive ways to lower CAR-T doses and improve control over exposure, but they do not remove on-target, off-tumor risk. PSMA is physiologically expressed at low levels on the luminal surface of proximal renal tubules and small-intestinal epithelium, whereas PSCA is present on normal urothelial and gastric epithelium and in tissues such as bladder, kidney and esophagus.

Alternatively, targeting antigens with more favorable safety profiles, such as CD126 (identified as commonly expressed across tumor types), has shown promise. CD126-specific CAR-T cells demonstrated effective tumor lysis with a reduced risk of cytokine release syndrome (CRS), offering a potentially safer therapeutic option (110).

A novel strategy to further minimize off-tumor effects employs synthetic Notch (SynNotch) receptors. This approach creates a conditional, logic-gated system: initial recognition of a primary tumor-associated antigen (TAA) by the SynNotch receptor induces expression of a CAR targeting a secondary antigen (111). Consequently, CAR-T cell activation only occurs upon engagement of *both* antigens on the target cell, significantly enhancing tumor specificity and sparing normal tissues (111). However, there are important PCa-specific hurdles to clinical translation. Key challenges for prostate cancer include the lack of systematic mapping of PSMA/PSCA co-expression across metastatic sites, marked inter- and intra-patient heterogeneity of PSMA expression on PSMA-PET, and the predominance of bone-predominant disease where obtaining paired biopsies for dual-antigen confirmation is difficult. These uncertainties raise a real risk that AND-gated circuits might spare normal tissues but fail to recognize a fraction of clinically relevant lesions, especially PSMA-high/PSCA-low bone metastases. As a result, synNotch or AND-gate CAR designs are probably best explored initially in patients with nodal or visceral metastases in which robust co-expression of the chosen antigen pair has been confirmed histologically, while patients with “pure” bone disease or very heterogeneous PET phenotypes may be less suitable for first-in-human trials.

ICANS is a distinct neurotoxicity of CAR-T therapy that presents with confusion, word-finding difficulty, delirium, somnolence, seizures, and—in severe cases—cerebral edema *(ASTCT)*. Pathophysiology implicates endothelial activation and blood–brain barrier (BBB) disruption, with IL-1 playing a central role (112); by contrast, IL-6 blockade does not reliably reverse ICANS, so corticosteroids are first-line (103), and IL-1 inhibition (e.g., anakinra) can be considered for refractory cases. Patients should undergo ICE score–based assessments every 8–12 hours with seizure prophylaxis as indicated, and tocilizumab should be reserved for concomitant CRS, not isolated ICANS. While ICANS has been prominent in hematologic CAR-T programs, prostate-directed trials remain small and variably report neurotoxicity; for example, in the phase 1 CART-PSMA-TGFβRDN study (NCT03089203), safety signals were dominated by CRS rather than neurologic events (113). Although ICANS has been less prominent than CRS in early PSMA/PSCA-directed CAR-T and TCE studies in mCRPC, the typical patient population is elderly with a non-trivial burden of cerebrovascular disease and baseline cognitive impairment. Key challenges for prostate cancer therefore include implementing intensive neurologic assessment in a frail, often anticoagulated cohort and balancing prophylactic corticosteroids or anti-cytokine strategies against the need to preserve T-cell function. In future studies, it seems reasonable to preferentially enroll patients without active CNS metastases or recent major neurologic events and to combine conservative step-up dosing with early ICANS-directed interventions, particularly when deploying high-potency BiTEs or multi-antigen logic-gated circuits.

## 6 Strategies to overcome immunosuppression in the tumor microenvironment

The prostate cancer tumor microenvironment (TME) exhibits pronounced immunosuppressive features that significantly hinder CAR-T cell therapy. Characterized by hypoxia, low pH, nutrient deprivation, and an abundance of immunosuppressive cells (e.g., TAMs, MDSCs, Tregs), the TME fosters the secretion of inhibitory molecules like TGF-β, PD-L1, and IDO, which collectively impair T-cell function. Here, we group the countermeasures into three approaches: (i) blocking dominant suppressive axes (e.g., TGF-β resistance and ROS detoxification); (ii) converting inhibitory cues into pro-survival/activation signals via synthetic receptors; and (iii) remodeling the stromal and vascular compartments. We summarize the mechanisms, representative evidence, and stage of development (preclinical vs. early clinical) to clarify translational readiness.

### 6.1 Block dominant suppressive pathways

#### 6.1.1 Blocking TGF-β signaling: engineering armored CAR-T cells

TGF-β is a key suppressor of cytotoxic T-cell function in the prostate TME. PSMA-directed CAR-T cells made insensitive to TGF-β—by expressing a dominant-negative TGF-β receptor II (dnTGFβRII) or by disrupting TGFBR2—regain effector function, show better expansion and favorable memory phenotypes, and improve tumor control in xenograft models. In a phase I study, TGF-β–insensitive PSMA CAR-T cells were feasible, showed dose-related expansion with manageable toxicity, and produced transient PSA declines (>30%) in some patients. These findings support continued development of TGF-β–resistant designs (65, 113).

#### 6.1.2 Detoxifying reactive oxygen species

Because high ROS further impairs CAR-T survival and killing, co-expression of catalase has been used to decompose hydrogen peroxide and reduce oxidative stress, thereby improving persistence and antitumor efficacy under harsh TME conditions in preclinical models. This strategy complements cytokine-axis interventions and may be layered with TGF-β resistance in future designs (114).

### 6.2 Bidirectional signal rewiring and combined checkpoint control

#### 6.2.1 ICR-enhanced PSMA-CAR-T (TGF-β → IL-7 rewiring)

The prostate TME provides abundant TGF-β, which dampens T-cell activity. Shao et al. engineered PSMA-CAR-T cells to co-express an immunomodulatory chimeric cytokine receptor that couples the TGF-β receptor ectodomain to the IL-7 receptor endodomain. When TGF-β binds, the receptor delivers IL-7-like pro-survival and activation signals. Compared with conventional PSMA-CAR-T cells, the ICR-enhanced cells produced more IFN-γ and TNF-α, showed stronger target-cell killing *in vitro*, and achieved deeper tumor control with longer survival in prostate cancer xenograft models. These data indicate that repurposing an inhibitory cue into an activating pathway can raise CAR-T potency in the prostate TME (115).

#### 6.2.2 Dual-modified PSMA-CAR-T (dnTGFβRII + truncated TIM-3)

Lei Tang et al. generated PSMA-CAR-T cells that co-express a dominant-negative TGF-βRII and a truncated TIM-3 to insulate the cells from two major inhibitory inputs. Under high TGF-β and TIM-3-ligand conditions, these dual-modified cells retained superior cytotoxicity and secreted higher levels of effector cytokines such as IFN-γ and IL-2. In mouse models, they suppressed tumor growth more effectively, prolonged survival, and reduced recurrence without apparent treatment-related toxicities. Concurrent blockade of TGF-β and TIM-3 therefore strengthens CAR-T performance in prostate cancer while maintaining a favorable safety profile (116).

### 6.3 Inducing TLS formation and vascular normalization: LIGHT-armored CAR-T cells

**Figure 3:**
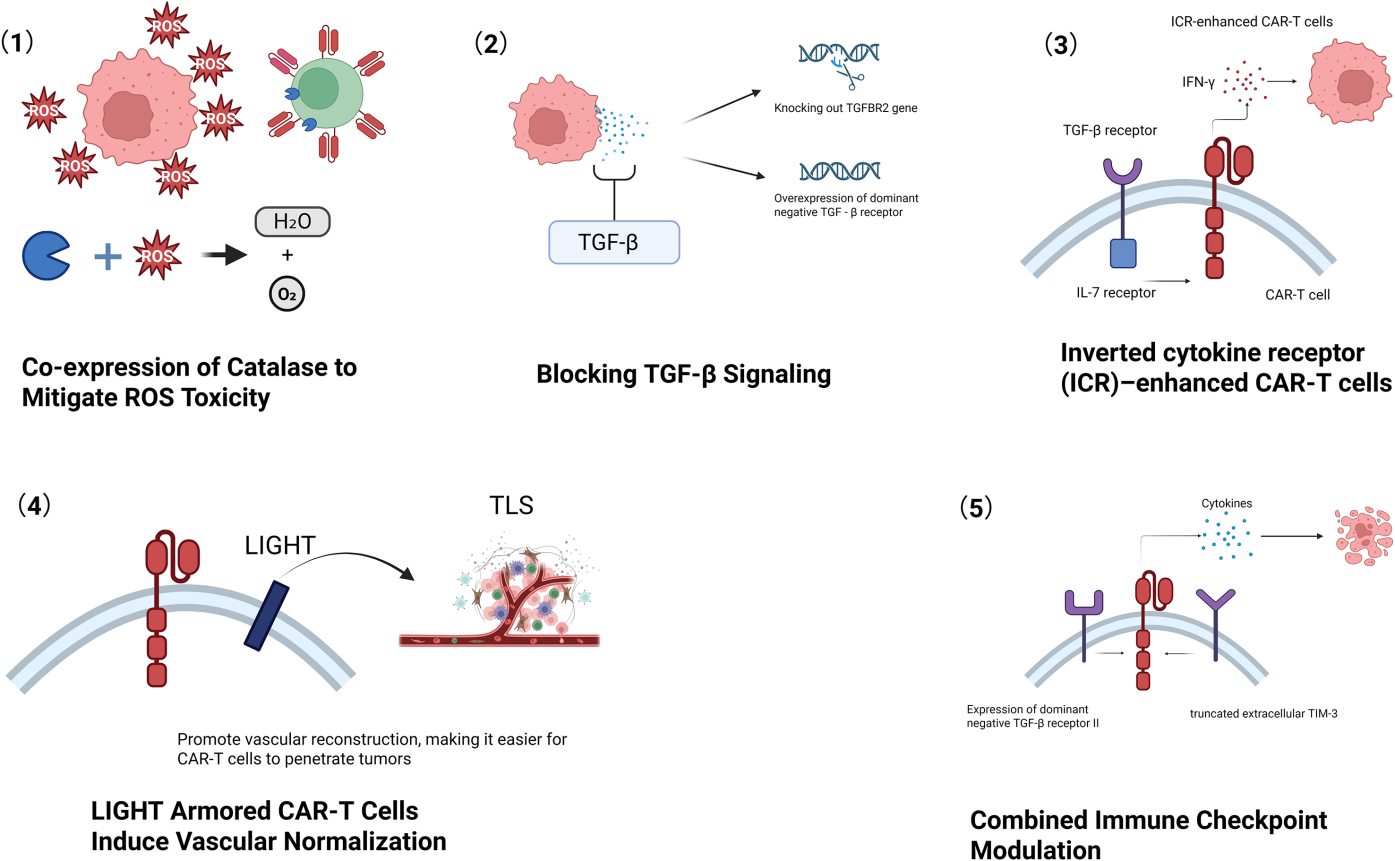
Strategies to overcome the suppressive tumor microenvironment in prostate cancer. This figure illustrates three engineered approaches to counteract key immunosuppressive barriers. (1) Antioxidant armoring: CAR‐T cells co‐express catalase (a reactive oxygen species [ROS]–degrading enzyme) to convert hydrogen peroxide into water and oxygen, thereby reducing oxidative stress and enhancing T‐cell survival and persistence under high‐ROS conditions. (2) TGF-β insensitivity: CAR‐T cells are rendered resistant to transforming growth factor–β (TGF-β) – for example by expressing a dominant-negative TGF-β receptor – which prevents TGF-β–mediated inhibition of T‐cell function and maintains cytotoxic activity. (3) ICR/switch-receptor engineering: an inverted cytokine receptor (ICR) converts inhibitory cytokine cues into pro-survival/activation signaling (e.g., TGF-β/IL-7 switch receptor). (4) LIGHT armoring: CAR‐T cells are engineered to secrete the TNFSF14 cytokine LIGHT, which promotes formation of tertiary lymphoid structures (TLSs) and normalizes tumor vasculature. LIGHT signaling (via HVEM/LTβR) recruits additional immune cells and upregulates homing chemokines, improving T‐cell infiltration. Together, these modifications reprogram the tumor niche – reducing suppressive cytokines and cells while improving vascular access – to enable more effective CAR‐T cell tumor eradication. (5) Combined inhibitory pathway modulation (e.g., dominant-negative TGF-β receptor II and truncated TIM-3 constructs) to reduce immunosuppression and improve cytokine production and cytotoxicity.

LIGHT-armored CAR-T cells co-express the cytokine LIGHT (TNFSF14) to reprogram the prostate TME. Engagement of HVEM/LTβR signaling upregulates homing chemokines (CCL19, CCL21), enhances T-cell infiltration and effector function, promotes tertiary lymphoid structure (TLS) formation, and normalizes aberrant tumor vasculature. These coordinated changes convert an immune-excluded niche into one that supports sustained CAR-T activity and endogenous antitumor immunity (117) (**Figure 3**).

## 7 Next-generation CAR-T engineering: overcoming exhaustion and enhancing persistence

Persistent antigen, hypoxia, and inflammatory cues in prostate tumors drive CAR-T dysfunction. Two cell-intrinsic edits have shown clear functional gains: dual knockout of PRDM1 and NR4A3 limited the exhaustion program, increased proliferation by ~2.3-fold, reduced PD-1/TIM-3 expression, and improved tumor control in NSG models (p<0.01); single-cell profiling placed these factors at the center of differentiation trajectories linked to long-lived, stem-like states (118).

However, each additional edit increases manufacturing and regulatory complexity: multiplex CRISPR engineering demands more stringent assessment of off-target effects, clonal composition and vector copy number, more elaborate release testing and, in practice, higher costs and a greater risk of batch failure than conventional single-edit autologous products. To show that strategies such as PRDM1/NR4A3 double knockout truly add value beyond standard CAR-T in prostate cancer, future studies will probably need to move beyond small, single-arm phase I cohorts towards randomized or at least parallel-cohort designs in which edited and non-edited CAR-T products targeting the same antigen are directly compared, with integrated correlative endpoints (CAR-T persistence, transcriptional state and TME remodeling) in addition to conventional PSA, radiographic and survival outcomes.

**Figure 4:**
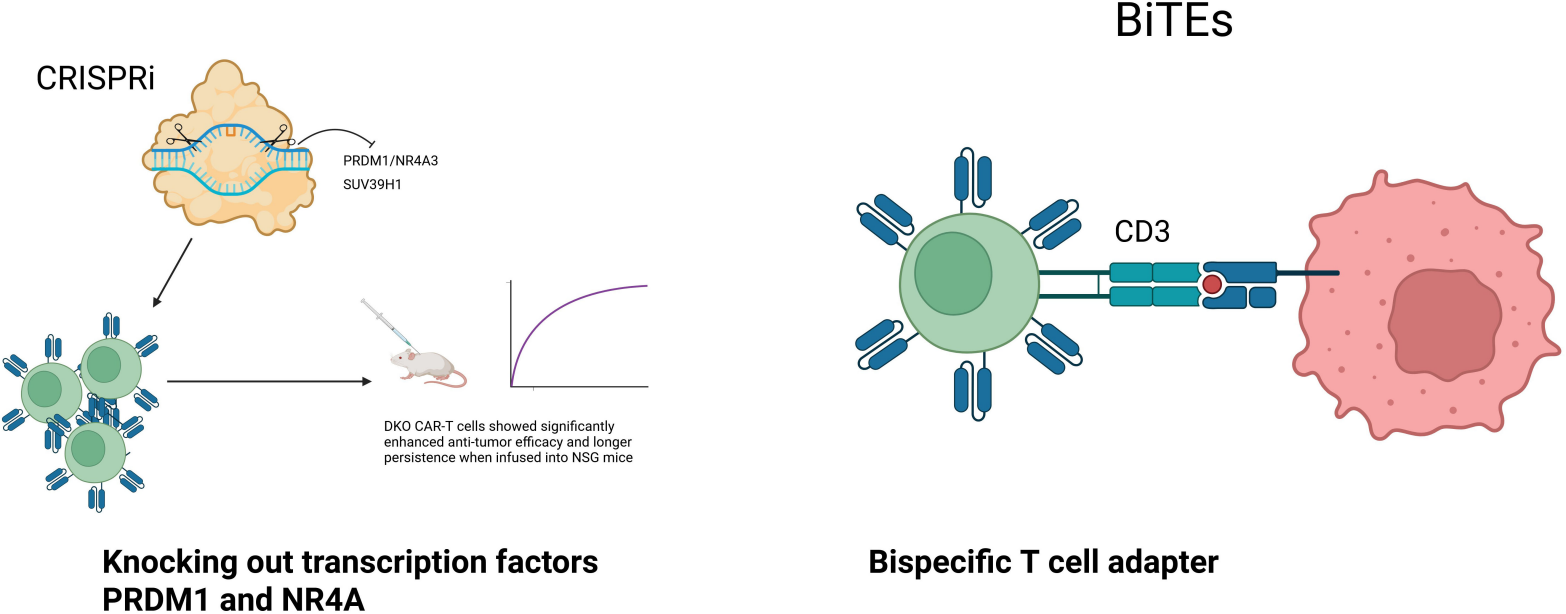
CAR‑T cell engineering and T‑cell engager strategies to enhance antitumor efficacy. CRISPR‑mediated disruption of exhaustion‑related transcriptional regulators improves CAR‑T cell function, memory phenotype, proliferation, and antitumor activity. Engineered bispecific T‑cell engager (BiTE) molecules bind CD3 on T cells and tumor antigens to facilitate T cell activation and tumor cell killing independent of MHC.

Deletion of the histone methyltransferase SUV39H1 lowered H3K9me3, preserved cytokine output (IFN-γ ~2.8×), enhanced expansion (p<0.001), cleared NALM6 tumors completely versus ~40% in controls, and maintained memory after repeat challenge; activity extended to PC3-PSMA xenografts, indicating applicability beyond hematologic models (119). Outside the CAR construct, a CD3-targeting bispecific antibody tightened immune synapses and improved killing of antigen-low targets (~70% vs 22% lysis), suggesting a practical add-on to reduce antigen-escape without altering the vector backbone (120). For translation, these edits can be introduced into lentiviral or transposon CAR products with CRISPR/Cas9; manufacturing should predefine vector copy number and RCL testing, off-target and translocation assays (amplicon-NGS or GUIDE-seq), and viability/expansion release criteria, with suicide switches or transient mRNA formats considered for early human studies. Finally, platform choices should be explicit: autologous T cells remain the reference, but universal T cells (e.g., TRAC/B2M-edited), NK, or macrophage effectors are credible alternatives when access or safety dictates; each carries specific risks (GVHD or rejection for allogeneic T cells, shorter persistence for NK, trafficking limits for macrophages) that should be addressed in trial design alongside prostate-specific barriers to infiltration and survival (**Figure 4**).

## 8 Combination therapy strategies

### 8.1 Synergizing CAR-T cells with immune checkpoint blockade

PD-1/PD-L1, CTLA-4, TIM-3, and LAG-3 checkpoint pathways promote T cell depletion in prostate TME; The purpose of combining immune checkpoint blockade (ICB) with CAR-T is to alleviate the poor efficacy of antigen binding, maintain effector function and persistence, and potentially expand intratumoral immunity through epitope diffusion (121). Integration can be achieved through systemic administration of PD-1/PD-L1 (± CTLA-4) antibodies, intracellular engineering of CAR-T cells to suppress or resist TME endocrine checkpoint inhibitors (e.g. dominant negative/PD-1 knockout), and by modulating multiple checkpoints to counteract compensatory pathways. In prostate cancer models, PD-1/PD-L1 blockade can enhance PSMA targeted CAR-T activity and improve tumor control, supporting biological synergy, compared to using either approach alone. However, prospective clinical data is still limited - early studies mainly evaluated CAR-T monotherapy with selective or exploratory use of ICB, and there are currently no randomized trials to determine the size of the effect, optimal timing, or patient selection in this group (122). Due to the potential amplification of inflammatory toxicity by ICB, experimental designs typically combine increasing CAR-T doses with pre-defined steroid/tocilizumab algorithms, or consider staging or delaying ICB, and closely monitor hematology/cytokines. The mechanism and principle support the pairing of ICB and CAR-T for the treatment of prostate cancer, but the clinical effectiveness has not yet been determined. Priority should be given to Phase I/II studies on safety run-ins to quantify benefits and identify responsive subgroups.

### 8.2 Augmenting CAR-T with conventional therapies

Here is currently no reliable data to test chemotherapy other than standard lymphocyte clearance in prostate cancer. Published studies include preclinical models and early/feasible clinical studies, in which chemotherapy is used for tumor reduction/bridging or biological regulation. Therefore, the current signal should be considered as a hypothesis rather than a certainty. In the xenograft model, PSMA directed CAR-T combined with low-dose docetaxel induced tumor regression, which was superior to simple cart cell infusion and chemotherapy alone, supporting the concept that selected chemotherapy can enhance CAR-T activity in PCa.

Early clinical trials of CAR-T in prostate cancer mainly evaluated CAR-T monotherapy, with chemotherapy primarily used for lymphocyte clearance or bridging. As of now, there is limited comparative data on the efficacy of cytotoxic chemotherapy, except for lymphocyte clearance, and the optimal drug selection, dosage, and timing have not yet been determined. Prospective controlled studies are needed to determine who benefits, how much benefits, and what the safety costs are.

Chemotherapy may enhance CAR-T through transient remission of immunogenic cell death, antigen presentation, and immunosuppression, but the clinical efficacy of this combination in PCa has not been confirmed. According to the preclinical principles of docetaxel+PSMA-CAR-T, reasonable, biomarker supported trials with clearly defined endpoints (such as PSA50, rPFS/OS) can be conducted in the future (66).

Beyond cytotoxic chemotherapy, several conventional modalities may serve as rational partners for CAR-T in prostate cancer. Short-course AR-axis therapy (e.g., enzalutamide) can transiently increase PSMA expression and may be used as priming or bridging, though clinical data with CAR-T are not yet available (123). Local radiotherapy can promote antigen release, dendritic-cell activation, and chemokine shifts that support T-cell trafficking; in practice it can function as bridging or peri-infusion priming, but prospective evidence specific to prostate CAR-T is lacking. PSMA radioligand therapy with ^177Lu-PSMA-617 is established in late-line mCRPC and can debulk disease before cell therapy (124); sequencing with CAR-T remains untested and should account for marrow reserve and timing around apheresis and conditioning. Overall, these approaches are hypothesis-generating and warrant controlled studies to define timing, selection, and safety.

## 9 Conclusion and outlook

CAR-based cellular immunotherapy for prostate cancer has shown biological activity and early clinical feasibility, but durable and reproducible clinical benefit remains to be established. In this review, we summarize the evolving landscape of prostate-associated targets and highlight engineering strategies aimed at overcoming key solid-tumor barriers—limited trafficking, antigen heterogeneity, and profound TME-driven dysfunction—together with emerging combination paradigms.

Current evidence supports a strong mechanistic rationale for approaches such as TME-resistance/rewiring, multi-antigen or logic-gated designs, and rational partnering with checkpoint blockade, radiotherapy, or other priming modalities; however, prospective clinical data in prostate cancer remain limited and have not yet demonstrated consistent improvements in clinically meaningful endpoints. Safety (including CRS, neurotoxicity, and on-target/off-tumor effects) and *in vivo* persistence continue to define the therapeutic window and should be addressed as rigorously as efficacy.

Near-term priorities include biomarker-enabled phase I/II trials with transparent safety run-ins, standardized pharmacodynamic readouts (expansion/persistence, exhaustion and activation markers, cytokine panels), and patient-centered endpoints, alongside explicit reporting of manufacturing and product quality attributes. These steps will clarify who benefits, by how much, and at what cost, and will guide the next generation of prostate-directed CAR therapies.
